# Effects of carbon ion beam irradiation on lung injury and pulmonary fibrosis in mice

**DOI:** 10.3892/etm.2013.881

**Published:** 2013-01-04

**Authors:** ZHENHUA WU, XINYU WANG, RONG YANG, YANG LIU, WEIPING ZHAO, JIN SI, XIAOFEI MA, CHAO SUN, YUANYUAN LIU, YONG TAN, WEI LIU, XIN ZHANG, CUIXIA DI, ZHENHUA WANG, HONG ZHANG, ZHONGXIANG ZHANG

**Affiliations:** 1 Institute of Modern Physics, Chinese Academy of Sciences, Lanzhou, Gansu 730000;; 2 Key Laboratory of Heavy Ion Radiation Biology and Medicine of Chinese Academy of Sciences, Lanzhou, Gansu 730000;; 3 Key Laboratory of Heavy Ion Radiation Medicine of Gansu Province, Lanzhou, Gansu 730000;; 4 School of Life Science, Lanzhou University, Lanzhou, Gansu 730000;; 5 Gansu Province Medical Science Institute, Lanzhou, Gansu 730000;; 6 College of Pharmaceutical Science, Shihezi University, Shihezi, Xinjiang 832002;; 7 Gastroenterology Department, The Yaojie Coal-Electricity Group Company General Hospital, Lanzhou, Gansu 730000, P.R. China

**Keywords:** carbon ion beam, irradiation, pulmonary fibrosis, lung injury, mice

## Abstract

Radiation-induced lung injury is a well-described complication of nuclear accidents, marrow-transplant pretreatment and thoracic radiotherapy. The mechanism is complex and no special therapy for it is available at present. To study radiation pulmonary injury following heavy ion radiotherapy for thoracic tumors, Kunming mice were randomly divided into 4 groups: normal control and 2, 4 and 6 Gy irradiation groups which underwent whole-body exposure to 235 MeV/u ^12^C^6+^ administered at the Heavy Ion Research Facility in Lanzhou (HIRFL). The pathological changes were observed by hematoxylin and eosin staining and the hydroxyproline (HP) content was assessed by spectrophotometry at months 1, 2, 3, 4, 5 and 6 after radiation exposure. In addition, the expression of tumor necrosis factor (TNF)-α and transforming growth factor (TGF)-β in the lung tissues was measured. The results showed that, compared with the control group, the lung tissue HP content was increased following irradiation but did not statistically significantly change after 4 months in the 4- and 6-Gy-treated groups. However, in the 2-Gy-treated group, the HP content was markedly increased between months 1 and 4 and decreased after month 4. The extent of the lung injury was significantly increased by the higher radiation dosages but was relieved in the 2 Gy group as the time since irradiation increased. The results also revealed that the levels of TNF-α were upregulated and reached a maximum at month 2, but decreased noticeably 2 months later in the experimental groups. The expression of TGF-β increased markedly in month 4 and was altered little in the 4- and 6-Gy-treated groups but decreased sharply in the 2 Gy irradiation group after month 4. These findings suggest that heavy ion radiotherapy for chest tumors causes lung injury to a certain extent, while there is likely to be little injury to lungs treated with <2 Gy, which provides scientific evidence for the use of heavy ion therapy for thoracic tumors.

## Introduction

The lung is one of the most radiosensitive organs, yet is occasionally irradiated as part of therapy programs for tumors of the lung, esophagus, breast and lymphatic system. Radiation-induced lung injury (radiation pneumonitis and radiation pulmonary fibrosis) is radioactive pulmonary damage which occurs when normal lung tissues are irradiated, such as in actinotheraphy for chest tumors, bone marrow treatment prior to transplantation and nuclear accidents, and is characterized by hypoxemia, non-cardiogenic pulmonary edema, low lung compliance and widespread capillary leakage. Radiation-induced lung injury is a common and critical problem that limits the doses that may be delivered in radiotherapy (1–3).

There are a number of possible etiologies for lung injury, but a clear cause for each patient may not usually be ascertained (4–5). The pathogenesis of pulmonary fibrosis remains unclear, although it has been widely accepted that the abnormal activation of certain inflammatory cells and cytokines in the lung is critical (6). In the initiating stage of the disease, numerous types of inflammatory cells, including macrophages, T lymphocytes, neutrophils and fibroblasts, are activated to release a variety of cytokines which contribute to cell aggregation. Subsequently, large amounts of inflammatory cytokines, such as tumor necrosis factor (TNF)-α, transforming growth factor (TGF)-β and interleukin-1β (IL-1β) are expressed by these cells. These cells and cytokines are responsible for abnormal airway tissue repair, which may lead to lung fibrosis (7–9). In the development of pulmonary fibrosis, proinflammatory cytokines and growth factors are considered to be significant in cell-cell and cell-matrix interactions (10–12).

Radiotherapy is a basic and important mode of treatment for nearly half of all tumor patients. Ionizing radiation kills tumor cells by inducing DNA damage, since non- or incorrectly repaired DNA results in lethal chromosomal aberrations which cause a loss of proliferative capacity. The formation of free radicals, such as the superoxide anion radical (O_2_^•−^) and hydroxyl radical (OH^•−^), is an unavoidable consequence of radiotherapy. These free radicals are extremely unstable and react rapidly with other groups or substances in the body, leading to cell or tissue injury. This may be explained by considering one of the numerous mechanisms by which oxidative stress causes damage by stimulating the free radical chain reaction. Free radicals activate TGF-β1, one of the most significant growth factors in the pathogenesis of fibrotic lung diseases, which promotes epithelial cell apoptosis (13–15).

Heavy ions, ionized atoms which are heavier than helium, are key components of cosmic rays and have linear energy transfer (LET) and high relative biological effectiveness (RBE) values. Heavy ions are also significantly more deleterious at the cellular and molecular level than low-LET ionizing radiation, including X-rays and γ-rays (16). Therefore, heavy ion irradiation is able to induce more unrepairable breaks in DNA damage than low-LET rays (17). It has also been demonstrated that heavy ion radiation produces chromosomal breakage and rearrangements and a greater degree of abnormal differentiation (18). Based on the excellent properties of heavy-ion beams, such as an energy deposition peak (Bragg peak) at the end of its range and an increased RBE within the peak, heavy ion radiotherapy is attracting growing interest worldwide (19) and is becoming one of the most promising therapeutic approaches for malignant tumors (20). However, heavy ion radiation not only destroys the tumor but may also potentially damage the normal tissue around the tumor. The objective of the present study was to evaluate the time, radiation dose, damage and repair effects of heavy ion radiation on lung injury and pulmonary fibrosis and provide experimental evidence for cancer therapy with heavy ions.

## Materials and methods

### Chemicals

Hydroxyproline (HP) assay kits were supplied by Nanjing Jiancheng Bioengineering (Nanjing, China). All other reagents were of analytical purity.

### Animals

SPF-class Kunming mice (male and female; age, 6–7 weeks; weight, 20±2 g) were provided by Lanzhou Medical College (Lanzhou, Gansu, China). The animals were housed in cages with *ad libitum* access to food and water and were kept in an environmentally controlled room (temperature, 23±1°C; humidity, 40±10%) with a 12 h light/dark cycle. All animal care and experiments were consistent with the Public Health Guide for the Care and Use of Laboratory Animals (National Research Council, 1996) and in accordance with protocols approved by the International Institutional Animal Care and Use Committee.

### Pretreatment for irradiation

A total of 144 mice, with an equal number of males and females, were randomly divided into 4 groups: a normal control (CK) group and 2, 4 and 6 Gy groups, with 36 animals per group. The CK group did not receive any treatment. The experimental groups received whole-body uniform carbon ion beam irradiation with 2, 4 or 6 Gy, at a dose rate of 0.5 Gy/min.

### Carbon ion irradiation and experimental design

The mice were positioned in a chamber fixed to the irradiation equipment at the Heavy Ion Research Facility in Lanzhou (HIRFL, Institute of Modern Physics, Chinese Academy of Sciences, Lanzhou, China). Each animal was placed in a cloth bag and underwent whole-body irradiation using a ^12^C^6+^ ion beam (235 MeV/u primary energy, ∼14.55 keV/*μ*m LET in water), at a dose rate of ∼0.5 Gy/min. Collimation of the beam to the irradiation location and the acquisition of data (preset numbers converted by doses of irradiation), were automatically performed by a microcomputer during the irradiation. The particle fluence was determined from an air-ionization chamber signal according to the calibration of the detector (PTW-UNIDOS; PTW-Freiburg Co., Wiesbaden, Germany). Sham-treated animals did not undergo irradiation.

### Lung index determination and histopathological examination

At months 1, 2, 3, 4, 5 and 6 after radiation exposure, the mice were weighed and then sacrificed by cervical dislocation (n=24, 6 per group). The lungs were excised immediately on an ice-cold plate, weighed and washed with physiological saline solution to prepare them for the subsequent experiments. Certain lung tissues were fixed with 10% formaldehyde in PBS buffer, dehydrated and embedded in paraffin. Next, 3–4-mm thick tissue sections were cut and stained with hematoxylin and eosin (H&E) for a histopathological observational study. Lung index = weight of lungs (mg) × 10 / body weight (g)

### HP measurement

Certain lung tissues were triturated and filtered through a 200-*μ*m pore mesh to remove debris or cell clusters, then used to analyze the HP levels. The lung HP content, considered as a biochemical index for the parenchymal collagen content, was measured in the lung tissue homogenate using a diagnostic reagent kit according to the manufacturer’s instructions (Nanjing Jiancheng Bioengineering) with analysis using a colorimetric method at 550 nm. The residual lung tissue homogenates were stored and frozen at −80°C until the biochemical analyses.

### Enzyme-linked immunosorbent assay (ELISA) for cytokines in lung tissues

The concentrations of TNF-α and TGF-β in the whole mouse lung tissue homogenates stored at −80°C at various time points were measured using a commercially available ELISA kit according to the manufacturer’s instructions (Nanjing Jiancheng Bioengineering Institute, Nanjing, China).

### Statistical analysis

Each experiment was repeated twice, with three samples for each treatment. The data were expressed as the mean ± standard error of the mean (SEM). Analysis of variance (ANOVA) with multiple comparison tests was used to determine the significance of differences between the groups. P<0.05 was considered to indicate a statistically significant difference. The correlation statistical analyses were performed using SPSS 11.5 for Windows (SPSS, Inc., Chicago, IL, USA).

## Results

### Morphological changes of the lungs

**Figure 1.**
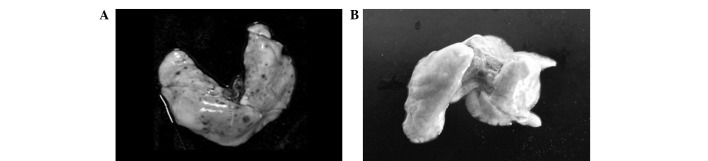
Appearance of fresh lungs from mice. (A) Irradiated lung (6 Gy) at month 4; (B) normal lung at month 4.

The changes in the appearance of the lungs of the mice undergoing carbon ion radiation began as acute lung injury followed by widespread acute pulmonary inflammation, indicated by increases in pulmonary edema. The lungs of the normal mice had a pink, soft, smooth and glossy surface, as well as fair elasticity upon touching. Compared with the CK group, the lungs of the mice irradiated with heavy ion beams were swollen with collapsed surfaces and poor elasticity. The irradiated lungs exhibited limited fibrotic involvement and appeared edematous, with noticeable hemorrhaging (Fig. 1).

### Changes in body weight of mice following carbon ion beam irradiation

As shown in Table I, during the 1st month following carbon ion beam irradiation (2, 4 and 6 Gy), the body weights of the mice decreased significantly due to the acute injury (P<0.05) compared with the CK group. Following month 1, the irradiated mice gradually regained weight, but remained significantly lighter than the CK group (P<0.05) during the whole 6-month experimental period. This result clearly indicated that ^12^C^6+^ irradiation caused a significant body weight loss compared with the normal mice, although the body weight increased continuously with time in all experimental animals.

### Changes in pulmonary index following carbon ion beam irradiation

The pulmonary index showed a marked increase (P<0.05 and P<0.01) after irradiation with various carbon ion beam doses with a clear dose-effect association compared with the CK group (Table II). Over the total experimental period of 6 months, the lung coefficient increased on the 1st, 2nd, 3rd and 4th months, but slightly decreased after the 4 month in each treatment group, while no evident changes were observed in the CK group. However, no statistically significant differences were observed between the various time points in each group (P>0.05).

### Lung tissue pathological examination

**Figure 2.**
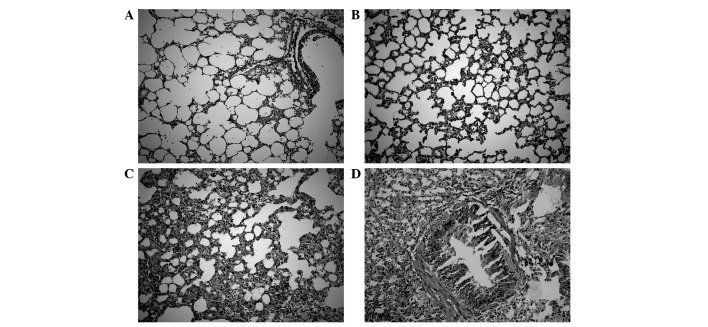
H&E staining of the histopathological changes in heavy ion radiation-induced lung injury and fibrosis. (A) Normal lung at month 6; (B) irradiated lung (2 Gy) at month 6; (C) irradiated lung (4 Gy) at month 6; (D) irradiated lung (6 Gy) at month 6. H&E, hematoxylin and eosin.

To evaluate the histopathological changes associated with heavy ion radiation-induced lung injury and fibrosis, the lungs of normal and ^12^C^6+^-irradiated mice were evaluated. The results showed that there were no pathological changes in the structure of normal lungs which consisted of a thin alveolar wall meshwork with few cells visible inside the alveolar spaces and no fibrosis. Significant histopathological changes and inflammatory reactions were observed in the heavy ion irradiated-groups, including the accumulation of numerous inflammatory cells in the alveolar spaces, extensive collagen deposition, heavily thickened alveolar walls and collapsed alveolar spaces, development of fibrotic lesions in the subpleural areas and focal honeycombing in the subpleural and peribronchial regions. The extent of the injury was significantly increased as the radiation dosage was increased compared with the CK mice. The injury was gradually relieved in the 2-Gy-treated group as the time since heavy ion irradiation increased (Fig. 2).

### HP content of lung tissue

**Figure 3.**
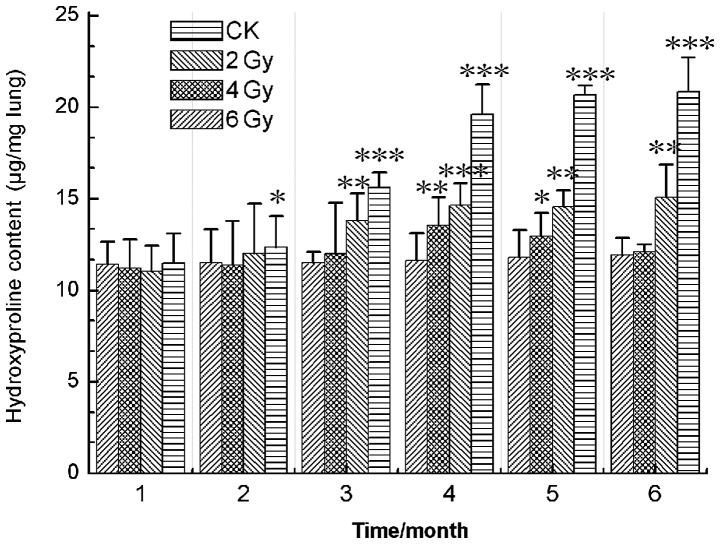
Effects of carbon ion beams irradiation on the hydroxyproline content of mouse lungs. ^*^P<0.05, ^**^P<0.01 and ^***^P<0.001, compared with the CK group. CK, normal control.

Pulmonary fibrosis was evaluated by measuring the HP content of the lungs, as an index of collagen accumulation. The results showed that the HP content of the lung tissue in the CK group was noticeably lower than those of the experimental groups and underwent no clear changes over the total experimental period of 6 months. The results also demonstrated that, compared with the CK group, the lung tissue HP content of the 4- and 6-Gy-treated groups was increased significantly (P<0.05, P<0.01 and P<0.001) after radiation exposure, although no statistically significant changes were observed after month 4. In the 2-Gy-treated group, HP content was markedly increased (P<0.05 and P<0.01) between months 1 and 4, but decreased following month 4 (Fig. 3). These results indicated that carbon ion beam irradiation induced lung injury and fibrosis.

### Expression of cytokines in the lung tissues

**Figure 4.**
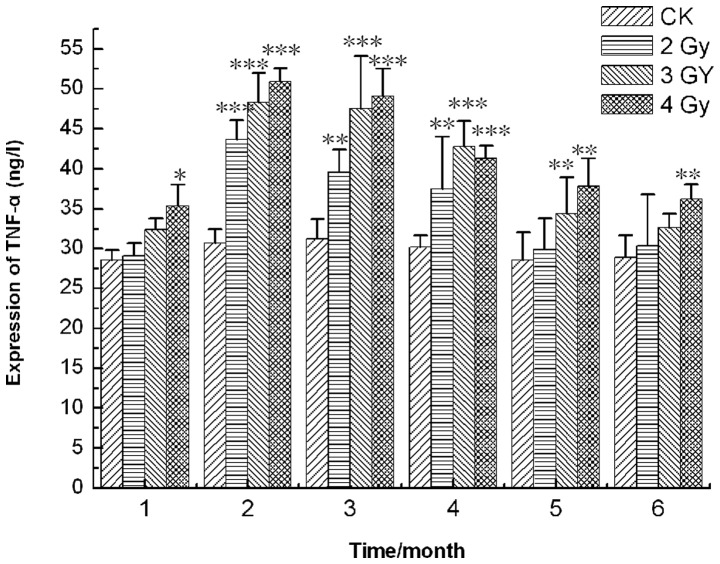
TNF-α concentration in ^12^C^6+^ irradiated lung homogenates. ^*^P<0.05, ^**^P<0.01 and ^***^P<0.001, compared with the CK group. TNF-α, tumor necrosis factor-α; CK, normal control.

**Figure 5.**
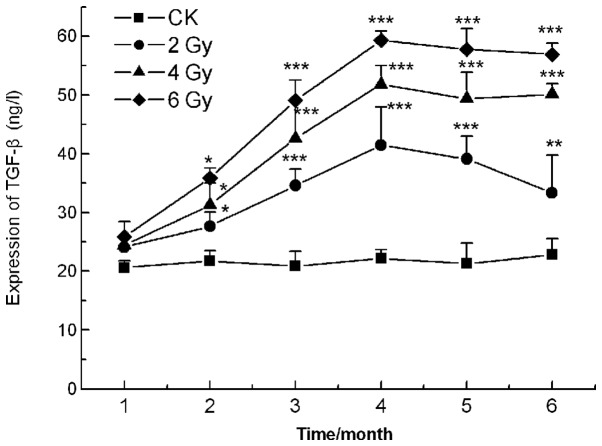
TGF-β levels in lung homogenates following carbon ion beam irradiation. ^*^P<0.05, ^**^P<0.01 and ^***^P<0.001, compared with the CK group. TGF-β, transforming growth factor-β; CK, normal control.

Inflammatory cytokines are significant in the pathogenesis and development of pulmonary fibrosis. The results revealed that the levels of TNF-α and TGF-β in all the carbon ion beam treatment groups were higher than those in the CK group. On the 2nd month following irradiation, the TNF-α content reached its maximum value, increasing to 43.7±8.6, 48.3±7.4 and 50.9±5.9 ng/l in the 2, 4 and 6 Gy groups, respectively, compared with 30.7±3.1 ng/l (P<0.001) in the CK group. After 4 months, the levels of TNF-α decreased noticeably in all experimental groups, but remained higher compared with the CK group (Fig. 4). The TGF-β levels increased markedly between months 1 and 4 in all irradiation groups, but subsequently changed little in the 4- and 6-Gy-treated groups and decreased sharply in the 2 Gy group (P<0.01, P<0.001; Fig. 5).

## Discussion

Heavy ion irradiation represents the best tool for the external radiotherapy of inoperable tumors and is now clinically used for tumor radiation therapy in Japan, America, Germany and China due to its outstanding physical and biological characteristics. Previous studies of heavy ions have demonstrated that, in comparison with LET radiation, such as electrons, X-rays and γ-rays, carbon ion beams are markedly stronger and more effective in radiotherapy at all of levels of biological organization (21). Heavy ions have been used in the treatment of various tumors, particularly radioresistant tumors mediated by hypoxia and those located near organs at risk, such as the brain, head, lung, liver, rectum and urogenital organs (22). However, our previous study results also showed that carbon-ion beam irradiation caused DNA strand breaks, cell apoptosis, lipid peroxidation, imbalance of antioxidant status, chromosome aberrations and inactivation of DNA repair enzymes (PARP-1) in mouse testes (22–23). At the same time as killing malignant cells in the treatment of cancer, carbon ion beams also kill normal cells near the tumors.

The lung, one of the most radiosensitive organs, is frequently irradiated as part of treatment programs for cancers of the lung, esophagus, breast and lymphatic system. Radiation-induced lung injury (radiation pneumonitis and radiation pulmonary fibrosis), a well-known side-effect of radiation following the post-operative chemotherapy or radiotherapy of breast tumors, is a common and critical problem that limits the radiation dosage that may be delivered in tumor therapy. At present, the etiology of this disease remains unknown. In the present study, the results coincided with previous studies and showed that the body weight of the mice decreased significantly in the 1st month and slightly increased after the 1st month following the carbon ion beam (2, 4 and 6 Gy) irradiation. This may be due to the acute radiation injury reducing appetite and disturbing the metabolic balance. The lung coefficient increased between the 1st and 4th months, but slightly decreased after the 4 month in each of the treatment groups, while no clear changes were observed in the CK group. It appeared that the tissue edema and congestion increased in the lung following carbon ion beam irradiation over the subsequent 4 months but decreased after this period.

The HP content of the lung tissue homogenates was closely associated with collagen accumulation and lung fibrogenesis, so the HP content of the lung tissue was used as an index of collagen accumulation and distinct marking for the clinical diagnosis of pulmonary fibrosis. In the present study, the lung tissue HP content increased significantly for 4 months after radiation exposure, but decreased in the 2-Gy-treated group and did not noticeably change in the 4- and 6 Gy-treated groups after 4 months. The histopathological changes also revealed that the extent of the lung injury was significantly increased when the radiation dosage was raised compared with the CK group and gradually relieved in the 2-Gy-treated group as the time since the heavy ion irradiation increased. A possible explanation for these findings is that the lung injury was less severe in the low dose irradiation group (2 Gy) than in the high dose groups and consequently easier to repair.

Radiation-induced lung fibrosis is a dynamic process characterized by the constant remodelling of fibrous tissue and long-term fibroblast activation (24–27). Previous studies have also suggested that pro-inflammatory and pro-fibrotic cytokines such as TGF-β and TNF-α, vascular injury, cellular adhesion molecules and oxidative stress are all vital in the development of radiation fibrosis (28–30). TGF-β is a well-known stimulant of extracellular matrix production by fibroblasts and has been demonstrated to be essential in the development of lung fibrosis (31–33). TNF-α is a pro-inflammatory cytokine that is critical in diverse cellular events and is also considered to be critical in the development of lung fibrosis. In the present study, the TNF-α content reached a maximum value in the 2nd month after radiation and decreased noticeably in all experimental groups after month 2. The levels of TGF-β increased markedly between months 1 and 4 in all irradiation groups, but decreased sharply in the 2 Gy irradiation group and changed little in the 4- and 6-Gy-treated groups from 4 months after irradiation.

A number of studies have indicated that the overproduction of reactive oxygen species (ROS) persisting after irradiation may be closely associated with tissue hypoxia and injury. ROS induce a cascade of cytokines and this is vital in the non-healing wound response that perpetuates lung injury (34–37). Therefore, the main task of future research is to regulate the content and levels of TGF-β and TNF-α by molecular biology methods such as biological modifiers, gene therapy and stem cell transplantation, to manipulate and mini-mise radiation-induced pulmonary fibrosis and injury.

The present study demonstrated that the carbon ion beam radiation led to acute lung injury, inflammation and fibrosis in mice in a time- and dose-dependent manner, similar to other forms of radiation. However, a certain degree of repair was observed at low doses with the lengthening of time after radiation. These results also indicated that whole-body heavy ion beam irradiation at lower doses (such as 2 Gy) in the normal lung is safer. The findings of the present study may offer references and experimental evidence for heavy ion radiotherapy.

## Figures and Tables

**Table I.** Changes in the body weight of mice following carbon ion beam irradiation.

	Body weight (g)					
Group	1 month	2 months	3 months	4 months	5 months	6 months
CK	30.3±2.1	35.5±5.0	39.1±4.7	41.6±1.9	44.8±1.4	44.5±3.2
2 Gy	25.1±3.4^a^	32.9±2.3	35.3±2.5	38.4±6.2	39.7±2.4^a^	42.3±2.7
4 Gy	24.2±1.1^a^	29.8±2.2^a^	33.7±1.6^a^	36.1±4.2^a^	37.3±1.6^a^	36.9±4.8^a^
6 Gy	23.9±1.9^a^	29.5±4.4^a^	34.1±1.3^a^	34.8±2.7^a^	37.6±3.3^a^	35.8±4.1^a^

Values are expressed as the mean ± SD, n=6.

a P<0.05, significant decrease compared with the CK group at the same time point. CK, normal control.

**Table II.** Changes in pulmonary index of mice following carbon ion beam irradiation.

	Pulmonary index					
Group	1 month	2 months	3 months	4 months	5 months	6 months
CK	7.31±1.9	7.29±3.1	7.28±0.5	7.34±2.4	7.40±0.9	7.37±0.8
2 Gy	8.23±0.4	8.60±1.4	9.12±2.4^a^	9.69±1.2^a^	8.24±3.1	8.05±2.0
4 Gy	8.93±2.3^a^	9.16±2.8^a^	9.97±0.6^a^	10.34±4.1^b^	10.06±2.2^a^	10.0±0.7^a^
6 Gy	9.08±0.5^a^	9.37±1.7^a^	10.06±2.3^a^	11.44±0.4^b^	11.13±0.9^b^	10.7±2.2^b^

Values are expressed as the mean ± SD, n=6.

a P<0.05,

b P<0.01, significant increase compared with the CK group at the same time point. CK, normal control.

## Abbreviations:

TNF-α: tumor necrosis factor-α;
TGF-β: transforming growth factor-β;
H&E: hematoxylin and eosin;
LET: linear energy transfer;
RBE: relative biological effectiveness;
HIRFL: Heavy Ion Research Facility in Lanzhou;
HP: hydroxyproline;
ELISA: enzyme-linked immunosorbent assay
